# Tuneable Hydrogel Porosity via Dynamic Tailoring of Spinodal Decomposition

**DOI:** 10.1002/advs.202504265

**Published:** 2025-06-26

**Authors:** Michael Halwes, Callum Vidler, Lilith Caballero Aguilar, Farzaneh Taromian, David R Nisbet, Melanie Stamp, Khoon S. Lim, Andrea J. O'Connor, David J. Collins

**Affiliations:** ^1^ Department of Biomedical Engineering University of Melbourne Parkville VIC 3010 Australia; ^2^ The Graeme Clark Institute The University of Melbourne Parkville Melbourne VIC 3010 Australia; ^3^ Aikenhead Centre for Medical Discovery St. Vincent's Hospital Melbourne VIC 3065 Australia; ^4^ Melbourne Medical School Faculty of Medicine Dentistry and Health Science The University of Melbourne Melbourne VIC 3010 Australia; ^5^ Faculty of Medicine and Health University of Sydney Sydney NSW 2006 Australia

**Keywords:** bioprinting, dynamic interface printing, GelMA, porous hydrogel, spinodal decomposition

## Abstract

Pores within hydrogel structures play a crucial role in fostering cell growth and tissue development. The creation and control of pore size and interconnectivity can be conveniently achieved with aqueous two‐phase emulsions. The decomposition of these emulsions into two separate phases can be controlled by carefully choosing the polymer components and solution conditions. Spinodal decomposition, a mechanism of phase separation, can result in a highly interconnected pore morphology, though controlling this process is difficult in practice, limiting its application for in vitro models. Here, a straightforward method is introduced for dynamically halting the phase separation of a gelatin methacryloyl and poly(vinyl alcohol) (GelMA‐PVA) polymer blend in the context of a biofabrication process based on dynamic interface printing (DIP). This is enabled by a novel approach based on the concerted application of acoustic mixing and photocuring to structure the pore size, orientation, and interconnectivity in hydrogels. This approach accordingly enables spatially addressable fabrication of 3D hydrogel architectures, with the potential to enhance the functionality of engineered tissues via tailored microenvironments.

## Graphical abstract

The authors introduce a straightforward method for dynamically halting the phase separation of a gelatin methacryloyl and poly(vinyl alcohol) (GelMA‐PVA) polymer blend in the context of a biofabrication process based on dynamic interface printing (DIP). The combined application of acoustic mixing and photocuring enables the tailoring of pore size, orientation, and interconnectivity in hydrogels.

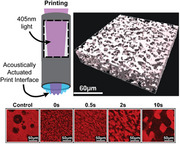

## 1 Introduction

Microphysiological systems are becoming increasingly popular for modelling human physiology in vitro, creating more complex and biomimetic environments for cell culture.^[^ ^1^ ^]^ Creating representative tissue‐level structures requires mechanical and chemical cues to guide cell spreading, migration, proliferation, and maturation. One such cue is the macroporosity (>Ø1 µm) of the hydrogels in which cells are grown.^[^ ^2^ ^]^ A variety of techniques exist for creating pores of different size, shape, and interconnectivity. Generally, these can be divided into kinetic methods, in which pores are created by arresting a dynamic process, or templating methods, in which porous structures are created from prefabricated templates, where kinetic methods typically have the advantage of being able to produce microfeatures in a scalable manner. Kinetic methods can involve generating gas bubbles within polymer precursors, inducing the separation of two‐phase emulsions, generating ice crystals within a polymer precursor or gel,^[^ ^3^ ^]^ or generating physical pores by means of a manufacturing process, e.g. electrospinning^[^ ^4^ ^]^ or 3D printing.^[^ ^5^ ^]^ The phase separation of aqueous two‐phase emulsions^[^ ^6^, ^7^ ^]^ through processes such as non‐solvent induced phase separation,^[^ ^8^ ^]^ thermally induced phase separation,^[^ ^9^ ^]^ or polymerization induced phase separation,^[^ ^10^ ^]^ has become a popular kinetic method due to its frequent biocompatibility and ability to be integrated with 3D bioprinting techniques.^[^ ^11^, ^12^, ^13^, ^14^ ^]^

Aqueous two‐phase emulsions arise from the repulsive interactions of a polymer with either its surrounding solvent or a second polymer species.^[^ ^15^ ^]^ Gelatin methacryloyl (GelMA) is often used as the bulk phase in cell culture scaffolds since it is biocompatible and includes both positively and negatively charged groups. The second polymer used as the porogen is often a neutrally charged polymer such as dextran, poly(ethylene glycol), or poly(vinyl alcohol) (PVA).^[^ ^16^ ^]^ Due to the way pores and interconnectivity depend on the underlying phase separation process, which is highly sensitive to constituent parameters, creating porous hydrogels with aqueous two‐phase emulsions is challenging. Phase separation depends on a combination of thermodynamic and electrostatic characteristics, including the polymer molecular weight, temperature, interfacial tension, and concentrations of the two components as well as pH, temperature, and the presence of ions in the solvent that can screen the electrical charges present on the polymer chains.^[^ ^17^, ^18^ ^]^ As a function of these parameters, a solution may separate into constituent phases via either nucleation and growth (metastable region of the phase diagram) or spinodal decomposition (unstable region). If the solution is metastable, i.e., if the solution falls within the binodal curve, defined by *dF/dx = 0*, where *F* is the Gibb's free energy of mixing and *x* is the composition of the solution, phase separation occurs via a nucleation mechanism. In this case, a local fluctuation in concentration must reach a critical radius before phase separation will occur, at which point droplets of one phase will continuously grow and coarsen^[^ ^19^ ^]^ (e.g., the growth of sugar crystals from a supersaturated solution). Alternatively, if the solution is unstable, i.e., if it falls within the spinodal curve defined by *d^2^F/dx^2^ = 0*, any local fluctuation will lead to phase separation, where these random local fluctuations simultaneously and constantly grow across the entire solution as described by the Cahn‐Hilliard equation.^[^ ^20^ ^]^ These distinct separation mechanisms consequently form distinct morphologies within the separated solutions.

Recent work has explored strategies for controlling the solution conditions, and hence the resulting porous architecture. Gan et al. used a microfluidic device to vary the concentrations of GelMA and polyethylene glycol in droplets to give varying pore sizes.^[^ ^21^ ^]^ As a result of the high interfacial tension between the two polymers solutions, the pores within the resulting microgels remained disconnected. Ben Messaoud et al. investigated the effect of pH on the pore morphology of a GelMA‐dextran system and found that, at pH values close to the isoelectric point of GelMA, the pores adopted an interconnected, bicontinuous morphology.^[^ ^12^ ^]^ This change in morphology was the result of the aqueous two‐phase system separating via spinodal decomposition, rather than nucleation and growth. By tuning the concentration of dextran in addition to the pH, they were also able to vary the length scale of the pores. The work highlighted the benefits of a bicontinuous morphology for encouraging cell growth and migration. In their case, however, the non‐physiological pH of the emulsion necessitated using post‐gelation cell seeding rather than concomitant encapsulation. When considering methods to scale up the production of these scaffolds, the need to equilibrate the pH of the gels across the scaffold volume following fabrication adds significant time delays, particularly for dense polymer networks. Creating accurate tissue models in a seamless workflow, therefore, would be substantially aided by an approach that allows direct cell inclusion during fabrication.

Despite being less investigated, shear can also affect phase separation mechanics and kinetics.^[^ ^22^ ^]^ For example, a rheometer was used to shear the pre‐polymer solution prior to photocuring, aligning the porogen phase along the flow direction, though a relatively large irradiation dosage was required in this GelMA‐dextran system (e.g., 120 s at 100 mW cm^−2^).^[^ ^23^ ^]^ Although the pore size and interconnectivity may be accordingly defined, this results in a homogeneous porous architecture throughout the gel. Further, these long gelation times and difficulty in implementing controlled flow present hurdles for utilization in a 3D bioprinting process, serving to further highlight the difficulty to date in practically utilizing aqueous two‐phase emulsions for biofabrication. Creating multiple pore sizes throughout a structure and tuning them in a deterministic manner, however, would allow for physiologically representative porosity gradients to be included.^[^ ^24^ ^]^

Here, we present an approach exploiting phase separation dynamics to tailor the porous architecture of hydrogels generated from aqueous two‐phase emulsions that is readily compatible with 3D bioprinting. Our technique involves homogenizing a solution containing two compatible polymers using acoustically driven flow and then allowing them to separate. By photocuring one or both polymer components of the solution, we can halt the phase separation process at specified time points, resulting in tuneable pore sizes and interconnectivity. Importantly, as the spinodal decomposition process control is physical rather than chemical, this is accomplished at physiological temperature, pH, and osmolarity. Moreover, staggering the photocuring timepoints across the exposure domain permits spatial tuneability in pore structure. To illustrate this approach and to demonstrate its potential value for biofabrication, we investigated a series of mixtures containing GelMA, polyvinyl alcohol (PVA), and a tyramine‐functionalized polyvinyl alcohol (PVA‐T), evaluating their phase separation dynamics for the design of macroporous hydrogels.

## 2 System Principles

**Figure 1:**
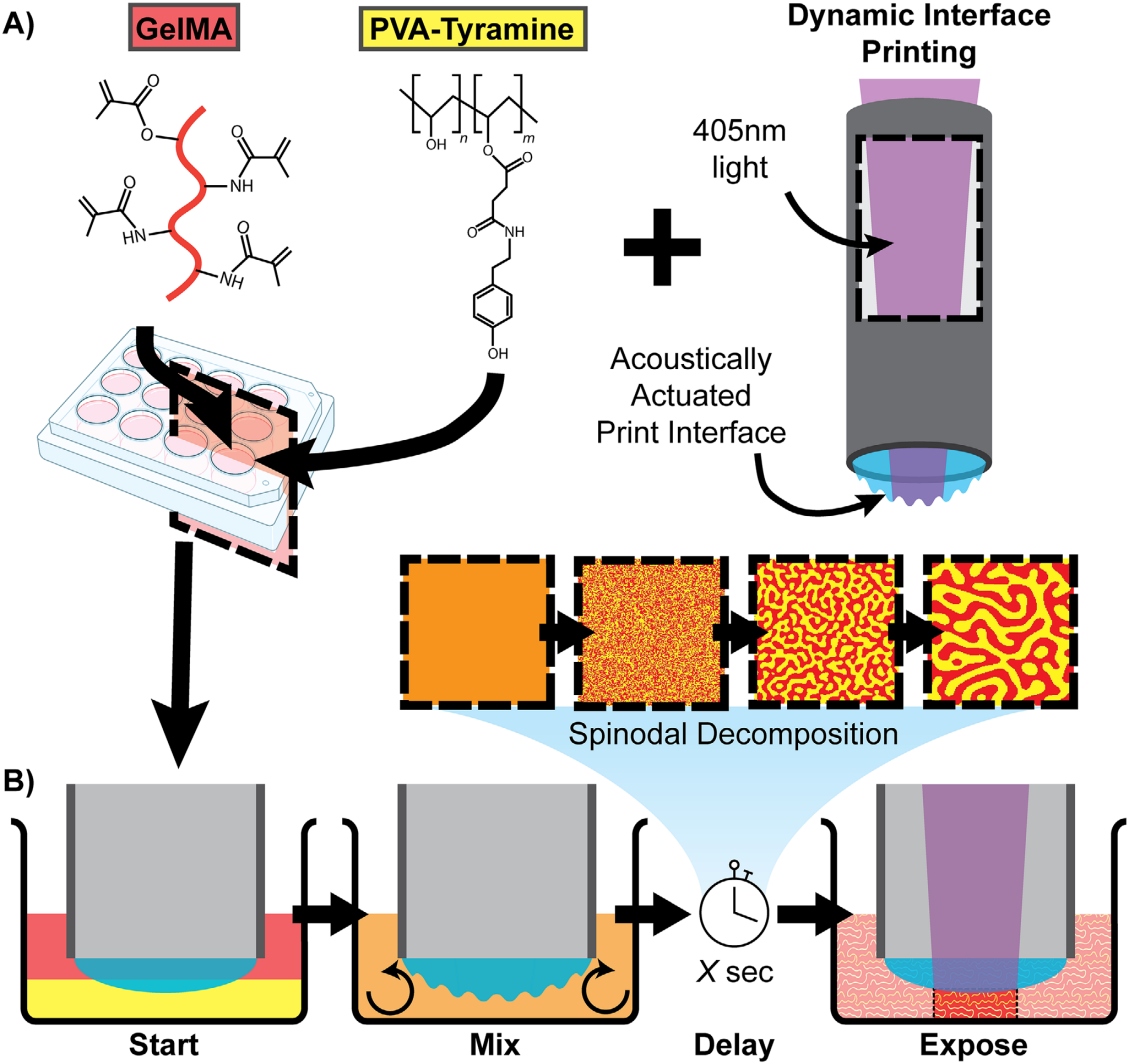
Schematic overview of the materials and fabrication process for the aqueous two‐phase emulsion hydrogels. A) Chemical structures of the two polymers used as well as a schematic drawing of the Dynamic Interface Printing print head. B) The gels were formed by first lowering the print head into the well containing the polymer blend, mixing the fluid using acoustic actuation, and then curing with 405 nm light after a prescribed delay period.

Here we demonstrate the ability to tuneably configure perfusable pore structures in macroporous hydrogels via an acoustically actuated two‐phase emulsion. Importantly, this approach is compatible with 3D printing, enabling the ability to spatially tailor porosity, and is achieved utilizing biocompatible materials at physiological pH. **Figure** **1** depicts the workflow followed to produce the macroporous hydrogel samples. Here the two polymer components, GelMA and PVA‐T, are combined in an aqueous solution along with a photoinitiator in a multi‐well tissue culture plate that is used as the vat for a novel biofabrication method, dynamic interface printing (DIP, Figure 1A).^[^ ^25^ ^]^ DIP is a recently introduced approach for rapid 3D fabrication that is distinguished by the use of a constrained air‐liquid interface as the printing surface, enabling this to be deformed and manipulated to enhance print functionality, including acoustic manipulation to generate displacements and capillary waves across the print interface. The print head of the prototype system is inserted into the fluid, trapping the volume of air within the print head to create a meniscus (Figure 1B). Projecting an acoustic signal into the print head causes vibrations to form on the surface of the meniscus, transmitting the mechanical energy into the aqueous solution and creating fluid flow. This oscillating flow creates local shear and thus mixes the two polymer components into a single‐phase solution. After the solution is homogenized, the acoustic mixing is halted and the solution comes to rest, at which point it begins to separate into two distinct phases via spinal decomposition, where the characteristic length scale of the phase separation increases with time according to Cahn‐Hilliard dynamics. Following a pre‐programmed delay period, 405 nm light is projected onto the meniscus surface to activate a photoinitiator within the polymer blend, crosslinking the polymer chains and thereby halting the phase separation process. This process is repeated for multiple delay periods and polymer blends to explore the impact of these parameters on pore size and orientation of generated macroporous hydrogels.

## 3 Results and Discussion

### 3.1 GelMA and PVA‐T Synthesis

Two batches of GelMA were synthesized and referred to as either “low DoF” GelMA or “high DoF” GelMA throughout the subsequent experiments. The absolute degrees of functionalization (DoF) of the two batches were 0.105 and 0.381 mmol_methacryloyl_/g_GelMA_, respectively, as measured by nuclear magnetic resonance (NMR). This corresponded to relative degrees of functionalization of 39% and 97%. The NMR spectra can be found in Figure S1 (Supporting Information). The absolute degree of functionalization is reported here as it is believed to allow for better comparability of GelMA between studies.^[^ ^26^ ^]^

Tyramine‐functionalized polyvinyl alcohol was synthesized via a two‐step reaction involving the addition of a carboxyl group (referred to hereafter as PVA‐COOH) followed by functionalization via 1‐ethyl‐3‐(3‐dimethylaminopropyl)carbodiimide/N‐hydroxysuccinimide (EDC/NHS) chemistry. Given the repeating structure of PVA, here the degree of substitution is reported as the percentage of repeating units functionalized with the given group. Although the PVA‐COOH showed a % conjugation of 2.75%, the PVA‐T only showed 0.55%, suggesting an incomplete conversion of carboxyl groups to tyramine groups. This could be explained by the low pH of the solution during the EDC/NHS reaction, the relative inefficiency of the EDC/NHS reaction in this context compared to the DCC/NHS reaction used previously,^[^ ^27^ ^]^ or steric hindrance caused by increased folding of the higher molecular weight PVA. The NMR spectra can be found in Figure S2 (Supporting Information).

### 3.2 Fabrication of Macroporous Hydrogels via Controlled Phase Separation

**Figure 2:**
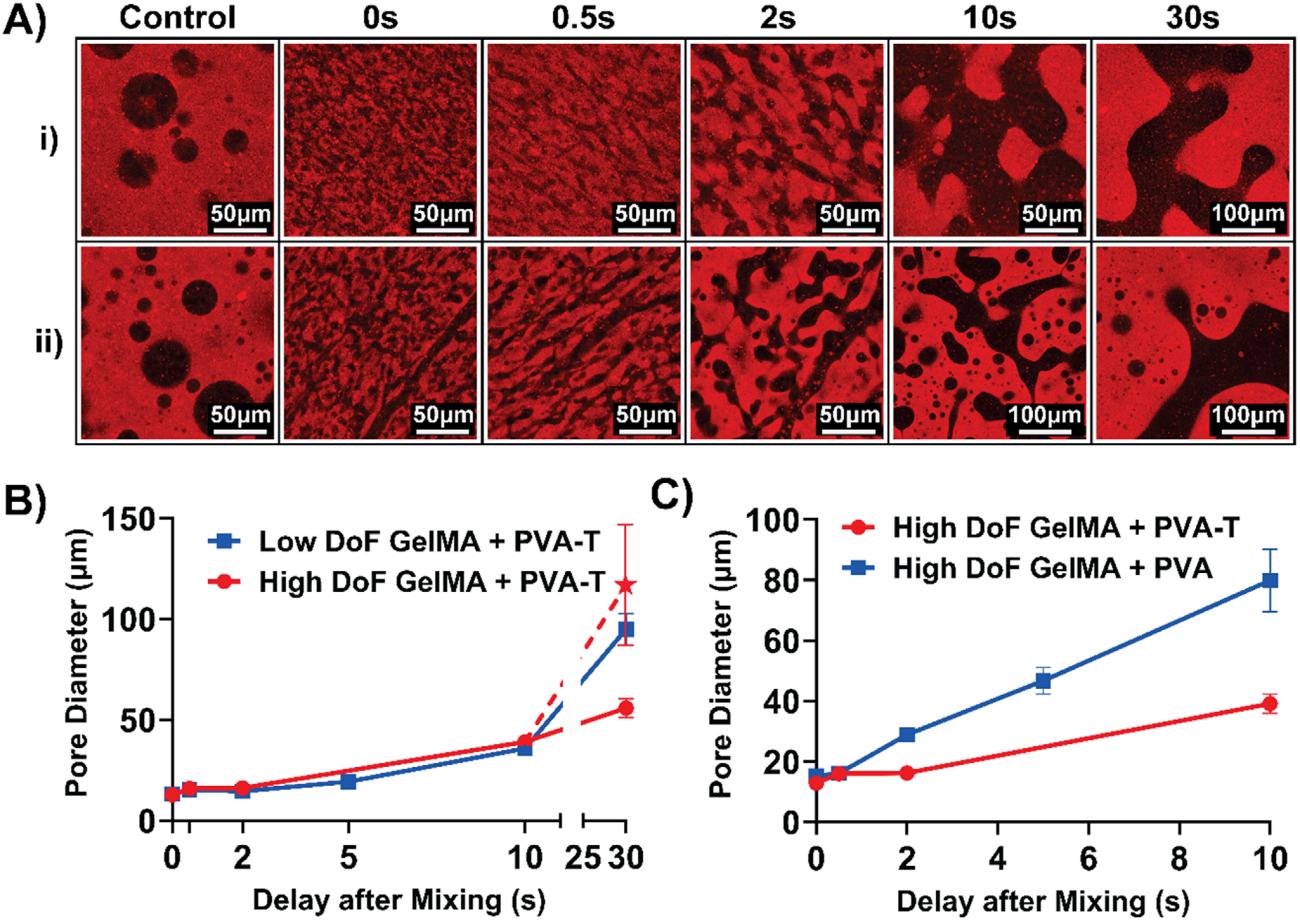
Results of the confocal analysis of pore size. A) Sample images taken from the confocal Z‐stacks at the time points tested. B) Sample 3D reconstruction of the pore network inside the hydrogel produced by the Imaris ML‐based segmentation. C) Sample 2D image taken from the distance transform of the reconstructed pore network. Here, a higher intensity corresponds to higher depth within the pore. D) Plot showing the calculated pore diameter for the low and high DoF GelMA + PVA‐T gels at various delay periods. The red star indicates the manually calculated pore diameter based off the confocal Z‐stacks, rather than the 3D reconstruction (see Results and Discussion). E) Plot showing the calculated pore diameter for the GelMA blends with PVA‐Tyramine versus PVA.

In all solutions, acoustically vibrating the print interface could be used to mix the two phases and create a more homogeneous solution than the control (**Figure** **2**A; Figure S3, Supporting Information). Using a ratio of 50 mg mL^−1^ GelMA to 30 mg mL^−1^ porogen (whether that porogen is PVA or PVA‐T), the solution consistently decomposed into a system in which the porogen created channels within the GelMA bulk phase. By increasing the delay between acoustic mixing and photocuring, the porogen phase continued to grow, resulting in larger pores.

Importantly, this separation only occurs with porogens with sufficient molecular weights (MW). During the time scales studied here, PVA‐T with low molecular weights (13–23 kDa) did not separate from GelMA sufficiently to form distinct pores (Figure S5, Supporting Information). This low MW PVA‐T was excluded from subsequent analyses so that clearer conclusions could be drawn from comparing the modified and unmodified high MW PVA mixtures.

Conversely, with a high MW (85–124 kDa) formulation, both PVA and PVA‐T formed a porogen phase inside the bulk GelMA phase (Figure S3, Supporting Information). However, the substantially higher exposure intensities and times required to cure the GelMA + PVA gels make PVA less ideal as an effective porogen choice for biofabrication of 3D cell culture scaffolds. This difference in required exposure intensity and time can be explained when considering two factors. Firstly, when using PVA‐T rather than PVA alone, the Tris(2,2'‐bipyridyl)dichlororuthenium(II) hexahydrate/sodium persulfate (Ru/SPS) photoinitiator system catalyzes the crosslinking of both methacrylate groups and tyramine groups, improving polymer interconnectivity and increasing the polymer hydrophobicity. Second, although we refer to the two phases as the GelMA “bulk” and PVA‐T “porogen” phases, it is important to note that the two molecules never completely partition themselves between the two compartments, even at thermodynamic equilibrium.^[^ ^15^ ^]^ It is more accurate to consider the bulk phase to be a “high GelMA, low PVA‐T” concentration phase and vice versa for the porogen phase. Figure 2A shows that aggregates of Rhodamine B (chemically bound to GelMA) are still visible within the porogen phase. Taken together, this suggests that PVA‐T acts to structurally reinforce the gel on both a macroscopic scale (by forming a network in the porogen phase) and the microscopic scale (by crosslinking with tyrosine groups present on the GelMA backbone). Moreover, given the negative effect that higher exposure intensities can have on the metabolic activity of encapsulated cells,^[^ ^28^ ^]^ PVA‐T appears to have significant biocompatibility benefits compared to PVA for future cell‐based studies in which cells may be directly encapsulated during printing.

### 3.3 Dynamic Control of Pore Size and Morphology

The maximum pore diameter was calculated based on the distance transform of the segmented 3D reconstruction of the porogen phase and plotted against the delay period between acoustic mixing and photocuring (Figure 2B,C). Both GelMA preparations have comparable pore sizes for both GelMA + PVA‐T mixtures across delay periods up to 10 s (Figure 2B). Here all values are expressed as mean ± standard deviation. The PVA‐T pore diameter starts at ≈13 µm for both GelMA batches (13.1 ± 1.1 µm for low DoF, 12.9 ± 0.8 µm for high DoF), growing to 35.8 ± 2.6 µm for the low DoF GelMA system and 39.1 ± 3.1 µm for the high DoF GelMA system after 10 s. After being allowed to separate for 30s, the two systems result in a 97.5 ± 9.7 µm pore diameter for the low DoF GelMA and 116.7 ± 29.7 µm for the high DoF GelMA.

Within each gel, the pore sizes are highly consistent, suggesting that 5 s of acoustic excitation is sufficient to uniformly mix the sample, regardless of delay period. One exception was the gel made from the high DoF GelMA and high MW PVA‐T when photocuring began 30 s after mixing. The sudden difference in pore diameter between the high and low DoF GelMA samples for the 30 s delay period was found to be caused by isolated droplets of GelMA that had become encapsulated within deeper regions of the PVA‐T phase in the high DoF GelMA samples (Figure S6, Supporting Information). While these GelMA droplets were correctly identified as such, they introduced errors when calculating the distance transform of the larger structure – the value reported by the automated measurement (represented by the filled circles in Figure 2B) was in fact lower than what was physically realistic. To account for this, the pore diameters for Z‐stacks of the 30 s delay gels for the high DoF GelMA were measured manually in ImageJ and marked separately on the plot with a star symbol. Although the standard deviation of these manual measurements was higher, the mean value supports the observed trend that the pores increased in size at comparable rates in both samples.

While the two GelMA + PVA‐T systems have similar pore sizes, their pore interconnectivity appears qualitatively different. This is especially the case for delay periods 10 s and longer, where the high DoF GelMA system appears to have a greater number of disconnected pores compared to the low DoF GelMA system. Compared to the control condition, both systems display a more interconnected morphology across all delay periods. Here, acoustically generated fluid shear homogenizes the two phases to the point that they begin to separate via spinodal decomposition. As time progresses, however, the GelMA and PVA‐T volume fractions within both the bulk and porogen phases must change since it is more thermodynamically favorable for the GelMA and PVA‐T molecules to be arranged separately than interspersed among one another. Additionally, more disconnected pores can be seen for longer delay periods in the confocal images (Figure 2A) and the 3D reconstructions (Videos S2 and S3, Supporting Information). This suggests that over time the system enters a state where it decomposes via nucleation, rather than spinodal decomposition (see Supporting Information). In this work, the goal was to demonstrate that spinodal decomposition could result in continuous pores with feature sizes relevant for cell culture scaffolds, rather than characterize the transition between spinodal decomposition and nucleation. Additional work is required to explore the thermodynamic properties of various polymer blends and assess their phase separation dynamics more directly, for example using real‐time microscopy or rheological evaluation.^[^ ^29^ ^]^

Whereas the GelMA degree of functionalization has only a minor effect on the generated pore sizes, functionalizing the PVA with tyramine had a significant effect on both the maximum pore diameter and the rate at which the pores grow (Figure 2C). This can be explained by the degree of intermolecular interactions between the GelMA and PVA or PVA‐T molecules. The higher degree of intermolecular interactions between tyramine groups on the PVA‐T and GelMA polymer chains acts to improve the miscibility of the two phases.^[^ ^30^ ^]^ By lowering the interfacial tension between GelMA and PVA‐T, the rate of phase separation is slowed.^[^ ^23^ ^]^

**Figure 3:**
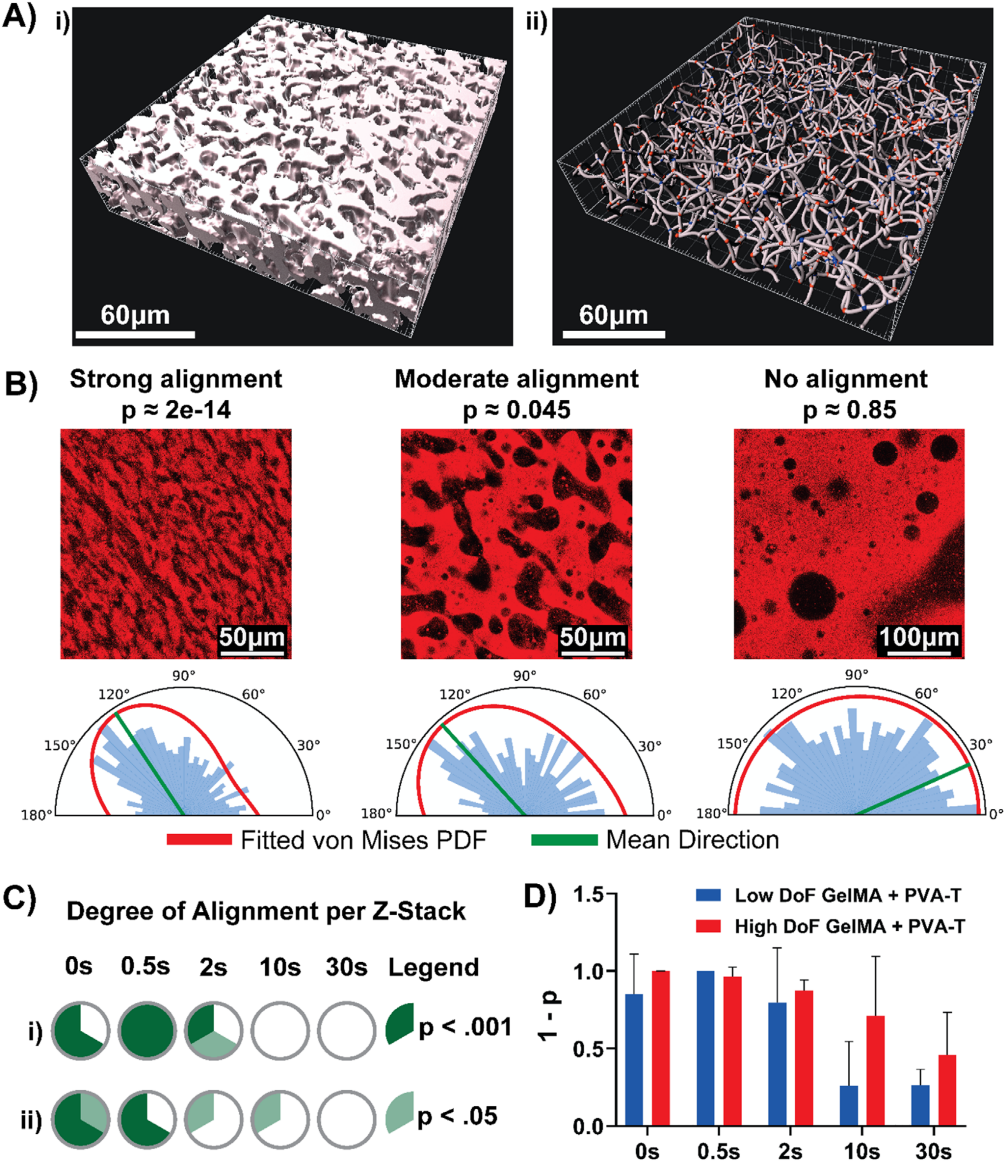
Image analysis results of pore orientation. A) 3D reconstruction of the Filament network produced by the Imaris software. B) Example semi‐circular rose plots showing the distribution of segment angles for different conditions. From left to right, High DoF GelMA + PVA‐T, 0 s delay; High DoF GelMA + PVA‐T, 2 s delay; High DoF GelMA + PVA‐T, 30 s delay. The plots provide a graphic representation of the degree of clustering around the mean direction as well as the von Mises probability distribution function. C) Sample‐wise results of the pore orientation analysis. Each circle for a given delay period is split into three sectors, each representing an individual Z‐stack dataset. Dark green indicates there was a high degree of orientation, light green indicates a slight degree of orientation, and white indicates no orientation per the Rayleigh z‐test for uniformity. D) Bar graph showing the average degree of pore orientation across the three Z‐stacks acquired for each gel. Error bars indicate the standard deviation of the p value.

Examining the orientation of pores within the gels, the image analysis workflow produces filament networks that match the segmented surface objects (**Figure** **3**A). The angles of segments making up these networks showed differing levels of clustering, which we classified as either showing strong, moderate, or no pore orientation based on the p values returned by the Rayleigh test for uniformity (Figure 3B). For each dataset, we plot the mean direction and von Mises probability distribution function, which is equivalent to the normal distribution for circular plots. The orientation of the pores is due primarily to the streaming flow generated by the acoustic mixing, where acoustic excitation of the printing interface results in differential flow patterns that are impacted by the excitation frequency. The influence of different acoustic parameters on flow velocity and direction has been analyzed in a previous study.^[^ ^25^ ^]^ Here, results indicate that residual momentum of the fluid may result in oriented pores when the gels are cured within 0.5 s of flow cessation. Importantly, the flow field is not uniform within the entire cross‐section of the print head, therefore the pore orientation in each Z‐stack was analyzed separately. These results accord with those of a recent study investigating the effects of shear on a GelMA/dextran two phase system, insofar as flow direction influences pore alignment, where the microstructure was shown to align in the direction of shear on a rheometer plate.^[^ ^23^ ^]^

### 3.4 Tailoring of Pore Size via Controlled Exposure Delays

**Figure 4:**
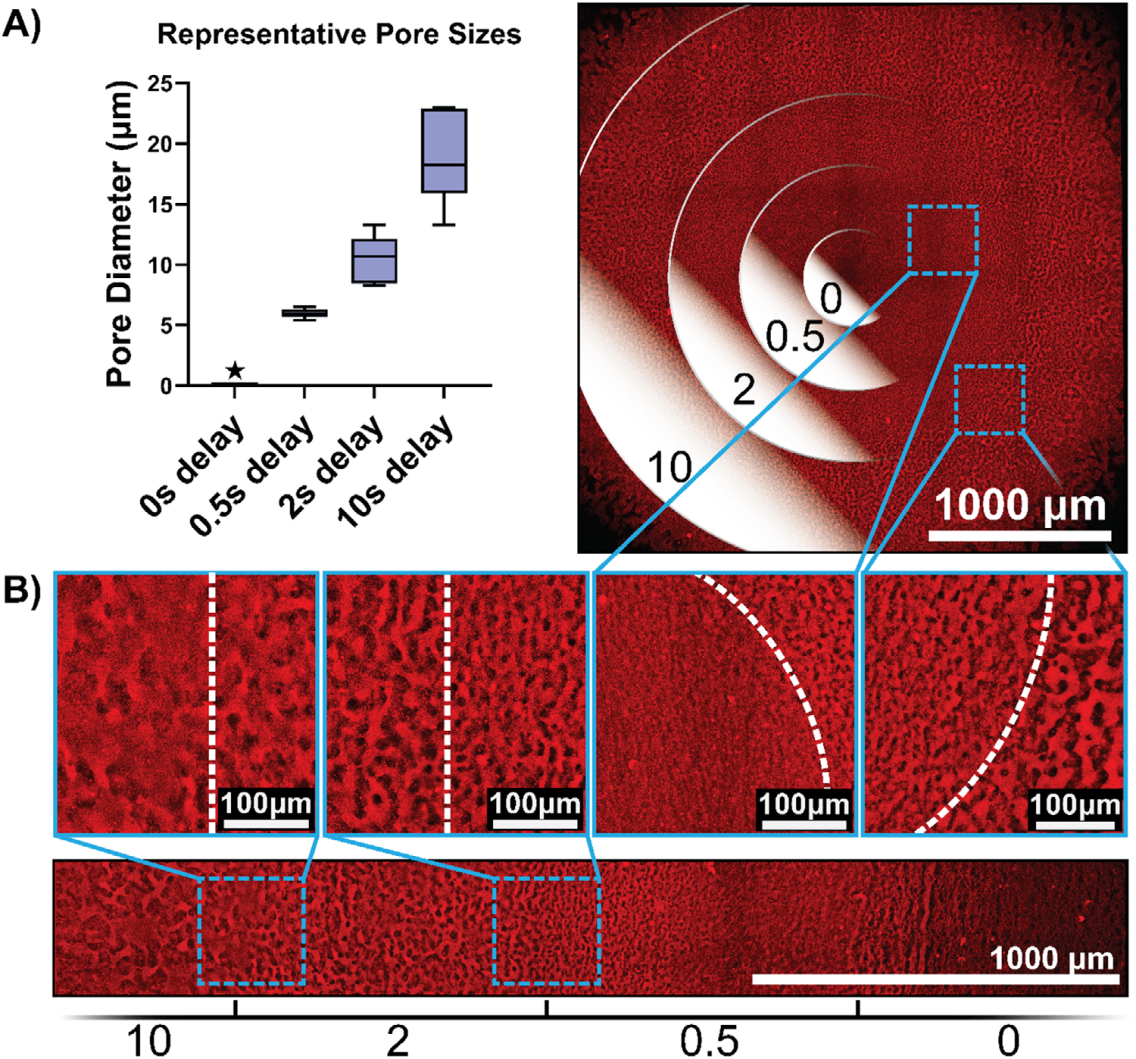
Confocal images showing the proof‐of‐concept gels containing multiple pore sizes. A) The 4 mm diameter circular gel, with the concentric rings indicating the delay period (in s) for that region. Inset images show the boundaries between neighboring sections (white dashed line). The box and whisker plot shows the diameter of pores measured manually in ImageJ. The 0 s delay is marked with a star icon, as no pores could be detected within that region. B) The 4 mm × 4 mm rectangular gel (cropped for presentation), with the delay periods marked underneath the image. Again, inset images show the boundaries between regions.

We further investigate whether subsequent exposures in a single layer could be used to spatially tailor pore size given the degree to which exposure timing can influence pore size. As a proof of concept, this was done for both a 4 mm diameter circular gel made up of concentric rings and a 4 mm × 4 mm square pattern made up of vertical bars (**Figure** **4**). Both geometries were successfully fabricated and exhibited distinct differences in pore size as a function of exposure delay. Despite the sequential exposure process and the different pore sizes, both gels maintained their structural integrity while being washed and handled prior to imaging. In both cases, the pores within the gel maintained an interconnected, bicontinuous morphology.

Based on these results, the DIP‐based tuneable porosity approach presented here is a valuable tool for tailoring the structural microenvironment, with significant potential for application in in vitro environments for 3D cell culture. There is broad interest in being able to tailor properties such as stiffness, topology, or chemical cues at length scales comparable to individual cells (10–150 µm).^[^ ^31^ ^]^ Doing so facilitates the curation of cellular responses such as spreading, migration,^[^ ^32^ ^]^ proliferation,^[^ ^33^ ^]^ and differentiation,^[^ ^34^ ^]^ though previous attempts to tailor hydrogels using biofabrication techniques have been limited by technical constraints such as the effective feature resolution of light‐based photolithography techniques.^[^ ^34^, ^35^ ^]^ To overcome these limitations, other recent work has utilized microfluidic mixers coupled to multiple material inputs to create gradients within structures fabricated in light‐based bioprinting systems.^[^ ^24^ ^]^ Microfluidic devices have also been used as reaction vessels in which 3D microgels can be fabricated via maskless photolithography.^[^ ^36^ ^]^ Aside from microfluidic techniques, high‐frequency acoustics have been used in many cases to tailor the microstructure of cell‐laden hydrogels. This includes, for example, modifying the polymer network of hydrogels to tailor their permeability and mechanical properties^[^ ^37^ ^]^ as well as positioning cells within extruded hydrogel fibers.^[^ ^38^ ^]^ The increased control over hydrogel structure, however, typically comes at the expense of the throughput of the technique, i.e., the rate at which structures can be produced.

Conversely, our approach allows us to create interconnected porous channels at scales from ≈10 to 120 µm, without introducing system features that limit the scale of the object being manufactured. Uniquely, this is also the first example where multiple porosities are patterned within a single material in a light‐based additive manufacturing process. For example, many microfluidic organ‐on‐chip models consist of multiple cell‐laden flow channels separated by a semi‐permeable membrane;^[^ ^39^ ^]^ it could be highly advantageous to tailor the porosity (and therefore permeability) of these membranes both within and between devices to match the permeability of the desired anatomical structures. Patterning multiple pore sizes within a single layer of a larger structure would be valuable in the context of vascular biology, where models of blood vessels covering multiple length scales would provide novel insights into tissue function.^[^ ^40^, ^41^ ^]^ Controlled pore orientation could also be a useful addition to scaffolds for nerve regeneration, where recent work has already shown the benefit of engineering the biochemical structure and mechanical properties of hydrogel materials.^[^ ^42^ ^]^ The possibility of adding colloidal particles to control the evolution of phase separation for more intricate structures^[^ ^43^ ^]^ could be explored in future work. Beyond cell‐based applications, tailoring the porosity of hydrogels could also assist in the rational design of medical devices and drug delivery vehicles for personalized medicine applications.^[^ ^44^ ^]^ For example, within the context of burn injuries, recent advancements have shown the benefit of hydrogel patches to improve healing by either conducting heat away from the wound site^[^ ^45^ ^]^ or releasing enzymes to reduce inflammation.^[^ ^46^ ^]^ Controlling the microstructure of these materials during the manufacturing process could further help to engineer their thermal conductivity or enzyme release profiles, further improving their clinical performance.

## 4 Conclusion

We have demonstrated a novel method based on acousto‐optic control of spinodal decomposition to tuneably structure the pore size, orientation, and interconnectivity in hydrogels created from aqueous two‐phase emulsions. By taking advantage of the transient phase separation mechanisms at play in the emulsion, both connected and disconnected pores can be created. When desired, the created pores remained consistent throughout the bulk of the hydrogels, with length scales in the range of 10–120 µm, matching the length scales of individual cells. The conjugation of tyramine to PVA further improved the manufacturability of the gels as well as the long‐term stability of the gels. Furthermore, the pore size can be deterministically patterned in each 2D layer by staggering the exposure of light.

Future work to integrate cells into the produced gels in specific organ‐on‐chip models could validate the utility of such an approach. Additionally, this study emphasizes the importance of selecting manufacturing methods that can leverage the inherent properties of the materials being produced. Here this approach is enabled by a novel biofabrication system termed dynamic interface printing (DIP) to induce acoustic mixing in viscous fluids directly at the site of fabrication. This approach has the potential to be broadly compatible with a wide range of polymer blends, both within and outside of biological applications. Going forward, additional materials or patterning modalities could be included to enable the formation of complex cellular microenvironments in tissue engineering applications.

## 5 Experimental Section

### Synthesis and ^1^H NMR Characterization of GelMA

Two batches of gelatin methacryloyl (GelMA) were synthesized based on a protocol by Zhu et al.^[^ ^47^ ^]^ For both batches, 250 mL of a 20% (w/v) gelatin solution (from porcine skin, Type A, 300 bloom, SigmaAldrich cat no. G2500) was prepared in 0.25 M carbonate‐bicarbonate buffer (SigmaAldrich cat no. C3041) while heating and stirring vigorously. NaOH (1 M) was added to raise the pH of the solution to 9.4 before slowly adding methacrylic anhydride (SigmaAldrich cat no. 276685). 4.690 mL (4.854 g) and 1.585 mL (1.640 g) of methacrylic anhydride was added dropwise for the high degree of functionalization (DoF) and low DoF batches, respectively. Then, the solution was left to react for 1 h at 55 °C before being quenched by reducing the pH to 7.4 using 36% HCl. The contents of the reaction vessel were diluted at a 1:2 ratio with MilliQ H_2_O before being dialyzed (12.4 kDa MWCO tubing, SigmaAldrich cat no. D0655) for 1 week, swapping the dialysate water at least once a day. Following dialysis, the solution was aliquoted into 50 mL centrifuge tubes, frozen overnight at −80 °C, and lyophilized for 1 week before being stored at −20 °C until use.

To prepare fluorescent GelMA samples for imaging, Rhodamine B was conjugated onto the GelMA (GelMA‐RB) using EDC/NHS chemistry.^[^ ^48^ ^]^ The same procedure was followed for both batches of GelMA. To start, 4.5 g of GelMA was dissolved in 50 mL of MES buffer (SigmaAldrich cat no. M3885) overnight at 37 °C. On the same day, 450 mg of Rhodamine B (SigmaAldrich cat no. R6626) was dissolved in 250 mL of MES buffer in a separate round bottom flask under continuous stirring, which was then covered in aluminum foil to protect the sample from light. 297 mg of 1‐Ethyl‐3‐[3‐dimethylaminopropyl]carbodiimide hydrochloride (EDC, ThermoFisher cat no. 22980) along with 544 mg of N‐hydroxysuccinimide (NHS, SigmaAldrich cat no. 130672) was added to the Rhodamine B solution and allowed to react overnight at room temperature. The next day, the GelMA solution was added to the Rhodamine B solution and allowed to react for 24 h at room temperature. The pH was then adjusted to 7.4 using 1 M NaOH to quench the reaction, and the contents were again dialyzed and lyophilized as above.

The DoFs of GelMA were analyzed with ^1^H NMR. 20 mg mL^−1^ solutions of GelMA and gelatin were prepared in D_2_O, and a 1 mg mL^−1^ nicotinamide (SigmaAldrich cat no. 72340) solution was used as an internal standard. By using nicotinamide as an internal standard, the total amount of cross‐linkable groups can be quantified, as had been validated in a previous study.^[^ ^26^ ^]^ Samples were prepared by combining 0.5 mL of GelMA or gelatin solutions and 0.2 mL of the internal standard. The samples were measured in a 400 MHz JEOL spectrometer (JEOL Ltd., Tokyo, Japan) at 37 °C. MestreNova (Mestrelab Research, Santiago de Compostela, Spain) was used to analyze the spectra. To quantify the absolute DoF (expressed in terms of mmol_methacryloyl_/g_GelMA_), the integrals of the methacrylate and methacrylamide group protons (in the 5.6–5.8 ppm range) were combined and normalized against the nicotinamide peak at 8.95 ppm. The absolute DoF was then calculated according to

(1) $$\mathrm{Do}F_{\mathrm{Abs}}=\frac{\mathrm{AU}C_{\mathrm{methacryloyl}}}{3H}*\frac{\mathrm{AU}C_{\mathrm{nicotinamide}}}{1H}*\frac{\mathrm{mmo}l_{\mathrm{nicotinamide}}}{mg_{\mathrm{gela}\mathrm{tino}\mathrm{rGel}\mathrm{MA}}}$$

See Claaßen et al. for a more thorough discussion of the absolute DoF calculation.^[^ ^26^ ^]^ For the relative DoF of the samples, the decrease in lysine in the GelMA samples was calculated relative to the unmodified gelatin. The lysine peak (2.97–3.1 ppm) in each sample was normalized against the benzene groups in the protein (7.2–7.45 ppm), and then the DoF was calculated^[^ ^49^ ^]^ according to

(2) $$\begin{array}{c} \mathrm{Do}F_{\mathrm{Rel}}=\left(1-\frac{\mathrm{AU}C_{\mathrm{lysine},\mathrm{GelMA}}}{\mathrm{AU}C_{\mathrm{lysine},\mathrm{gelatin}}}\right)\;*100 \end{array}$$

### Synthesis and ^1^H NMR Characterization of PVA‐Tyramine

PVA‐Tyramine was synthesized based off a two‐step synthesis protocol from Lim et al.^[^ ^27^ ^]^ Briefly, polyvinyl alcohol (PVA, 87–89% hydrolyzed, MW 85–124 kDa) was first modified to include a carboxyl group (PVA‐COOH) for subsequent functionalization with tyramine (PVA‐T). To add the carboxyl group, 10 g of PVA was dissolved in 85 mL DMSO (SigmaAldrich cat no. D2438) at 60 °C under a nitrogen atmosphere while stirring. Then, 450 mg succinic anhydride (SigmaAldrich cat. no. 239690) and 610 µL triethylamine (SigmaAldrich cat. no. 90340) were added, and the reaction was allowed to proceed for 24 h at 60 °C. The products were then precipitated in 500 mL ethanol and vacuum filtered before being redissolved in 200 mL boiling MilliQ water. The resulting solution was then purified via dialysis for 4 d, swapping the dialysate twice a day, before being freeze dried for an additional 4 d. Before proceeding to the tyramine functionalization, the degree of carboxyl group conjugation was measured using ^1^H NMR. The measured conjugation was ≈2.75% (see Supporting Information), and this value was used to calculate the required masses of EDC, NHS, and Tyramine HCl in the subsequent reaction.

The PVA‐COOH was then functionalized with tyramine using EDC/NHS chemistry, instead of the DCC/NHS method previously used.^[^ ^27^ ^]^ To start, 3.75 g PVA‐COOH was dissolved in 100 mL of 50 mm MES Buffer at 70 °C under nitrogen atmosphere while stirring. The pH of the solution was adjusted to 4.7 using 36% HCl and allowed to cool to room temperature before 1.335 g NHS and 0.7157 g EDC were added. The reaction was allowed to proceed for 45 min before the pH was raised to 5.4 using a 1 M sodium bicarbonate buffer. At this point, 800 mg Tyramine HCl (SigmaAldrich cat. no. T2879) was added, and the reaction proceeded at room temperature for 24 h. The products were then precipitated in 1 L acetone, redissolved in MilliQ water, and purified via salt‐gradient dialysis. For this, consecutively lower concentrations of NaCl (150–0 mm in 25 mm steps) were prepared in the dialysate, swapping once per day. The dialysis proceeded for an additional 4 d afterward, swapping the dialysate once per day. The purified products were then freeze dried and stored at −20 °C until use.

To analyze the degree of conjugation of the functional side groups, 20 mg mL^−1^ solutions of PVA‐T, PVA‐COOH, and base PVA were prepared in D_2_O. An additional 20 mg mL^−1^ sample of a low MW (13–23 kDa) PVA‐T was also prepared for comparison. The ^1^H NMR measurement settings matched those described above for the GelMA analysis. To calculate the degree of carboxylation, the peak at 2.4–2.65 ppm corresponding to the four contributing methylene protons of the carboxyl group was normalized against the peak at 3.4–4.2 ppm corresponding to the one methylene proton of the polyvinyl alcohol repeating unit backbone, with

(3) $$\begin{array}{c} \mathrm{DoC}\left(\%\right)=\left[\frac{\mathrm{AU}C_{\mathrm{methylene},\mathrm{COOH}}}{4}\;*\frac{1}{\mathrm{AU}C_{\mathrm{methylene},\mathrm{PVA}}}*100\right]\; \end{array}$$

To calculate the degree of tyramine functionalization, a similar calculation was performed, this time using the peak at 6.7–7.2 ppm corresponding to the four contributing protons present in the tyramine aromatic group, with

(4) $$\begin{array}{c} \mathrm{Do}F_{\mathrm{Tyramine}}=\frac{\mathrm{AU}C_{\mathrm{aromatic},\mathrm{Tyr}}}{4}\;*\frac{1}{\mathrm{AU}C_{\mathrm{methylene},\mathrm{PVA}}}*100 \end{array}$$

The NMR spectra of the measured samples can be found in the Supporting Information.

### Interfacial Tension Measurements

To quantify the shift in interfacial tension introduced by the functionalization reactions, samples of unmodified PVA, high MW PVA‐T, low MW PVA‐T, high DoF GelMA, and low DoF GelMA were measured via the pendant drop method using a DataPhysics OCA 25 tensiometer (DataPhysics Instruments GmbH, Germany).^[^ ^50^ ^]^ 30 mg mL^−1^ solutions of the PVA samples and 50 mg mL^−1^ solutions of the GelMA samples were prepared in MilliQ H_2_O, matching the concentrations used in later experiments. For each sample, three repeats were measured. The differences in mean interfacial tension were analyzed using one‐way ANOVA in GraphPad Prism (GraphPad Software Boston, USA). Results can be found in Figure S7 (Supporting Information).

### Rheological Characterization

Viscosity measurements were taken to illustrate how the rheological properties of the mixtures might have affected the phase separation dynamics. For this, samples of unmodified PVA, high MW PVA‐T, and low MW PVA‐T were prepared, both individually and in combination with the high DoF GelMA. Shear ramp measurements from 1 to 1000 s^−1^ were taken on an Anton Paar Rheometer (Anton Paar GmbH, Graz, Austria) using a 15 mm diameter truncated cone plate. Each sample was measured three times, using 40 µL of sample per measurement. Results can be found in Figures S8 and S9 (Supporting Information).

### Fabrication of Porous Gels using Acoustic Mixing

Samples of the aqueous two‐phase emulsion gels were prepared via acoustic mixing in a prototype biofabrication system (Figure 1A).^[^ ^25^ ^]^ For all samples, the two‐component prepolymer mixture comprised either the high or low DoF GelMA‐RB combined with either unmodified high MW PVA, low MW PVA‐T, or high MW PVA‐T. The respective concentrations of the two polymer components remained the same across all samples: 50 mg mL^−1^ for GelMA‐RB, and 30 mg mL^−1^ for PVA‐T. In all samples, the photoinitiator was a combination of 1 mm Tris(2,2′‐bipyridyl)dichlororuthenium(II) hexahydrate (Ru, SigmaAldrich cat. no. 224758) and 10 mm sodium persulfate (SPS, SigmaAldrich cat. no. S6172). All components were initially dissolved in DPBS prior to being combined. All concentrations listed were the final concentrations of the respective components. Stock solutions of 200 mg mL^−1^ GelMA‐RB, 100 mg mL^−1^ PVA‐T, 10 mm Ru, and 100 mm SPS were used to create each mixture. 2 mL of each mixture was prepared to manufacture the gels for that mixture.

To create the pre‐polymer mixtures, the GelMA and PVA were first combined, diluted with DPBS, and vortexed for 30 s. The Ru and SPS were then added, and the sample was covered in aluminum foil to protect from light. The sample was then placed in a water bath at 37 °C to ensure the mixture did not thermally gel. Immediately prior to being loaded into the biofabrication system, the samples were vortexed for an additional 30 s and repeatedly pipetted using a 1 mL pipette tip 10 times. The entirety of the mixture was then pipetted into one well of a glass coverslip‐bottom, methacrylate‐treated 12‐well plate (Cellink cat. no. D16110025295). The well plate was then loaded into the enclosure of the biofabrication system, whose internal temperature was maintained at 37 °C.

To begin, the hollow cylindrical print head was first immersed in the pre‐polymer mixture to establish a trapped meniscus (Figure 1B). This meniscus represents the interface at which the gels were cured using 405 nm light. Prior to curing, however, an acoustic signal was utilized for 5 s which caused the meniscus to vibrate (Video S1, Supporting Information). These vibrations drive fluid flow in the mixture to actively mix and homogenize the solution. After 5 s of acoustic mixing, a preprogrammed delay elapses before the photocuring occurs. For all gels, the exposure pattern was a 4 mm diameter circle. Different acoustic parameters were used for the mixtures to create fluid flow, which depended on the viscosity of the mixtures (Figures S8 and S9, Supporting Information). Acoustic frequencies of 25 and 20 Hz were used for the low DoF GelMA and high DoF GelMA mixtures, respectively, with amplitudes of 3.339 ± 0.013 kPa and 4.306 ± 0.043 kPa (mean ± standard deviation), as measured by a MEMS pressure sensor (Mitsumi MMR920C04 I2C Board) connected to the air cavity within the print head. These values were selected based on previous particle image velocimetry measurements exploring the ability of acoustic stimulation to generate fluid flow at the print head.^[^ ^25^ ^]^ The high flow velocities observed in those studies (>10 mm s^−1^) as well as the small standard deviation of the observed pore sizes (see Results and Discussion) indicated that 5 s of mixing was sufficient for consistent analysis. Once the photocuring ceases, the print head was removed from the prepolymer mixture, which was then transferred to the next well for the subsequent gel.

For each pre‐polymer mixture, the first gel manufactured was a control, meaning no acoustic mixing was performed. Here the print head was immersed in the solution, the meniscus was established, and photocuring commenced. The subsequent gels had preprogrammed delay periods ranging from 0 s (i.e., no delay) to 30 s between acoustic excitation/mixing and photocuring. For prepolymer mixtures containing PVA‐T, photocuring was performed at 140 mW cm^−2^ for 1 s. For mixtures containing PVA, photocuring was performed at 350 mW cm^−2^ for 5 s. The higher photocuring intensity and duration was due to the lower number of chemical groups available for crosslinking when using PVA, compared to PVA‐T (See Results and Discussion). After each gel was cured, it was rinsed twice with 2 mL of DPBS. All gels were subsequently incubated overnight in DPBS at 37 °C before being imaged.

### Confocal Microscopy

All gels were imaged on a Nikon AR1 confocal microscope (Nikon Corporation, Tokyo, Japan) using a 555 nm excitation laser. The gels were imaged under swollen conditions using a 20x, 0.75NA objective. For each gel, 3 separate Z‐stacks were acquired at random XY locations within the gel, at starting Z positions ≈50 µm above the glass coverslip bottom of the well plate. Depending on the size of the pores observed, the acquired Z‐stacks were either 40 µm thick with a 0.95 µm step size and a 1024 × 1024 px field of view or 80 µm thick with a 2 µm step size and a 2048 × 2048 px field of view.

### Image Analysis

To analyze the pore structures observed within the gels, including pore dimensions and interconnectivity, the confocal Z‐stacks were processed using Imaris v10.2.1 (Oxford Instruments plc, Abingdon, UK). Prior to being converted to the Imaris file format, the Z‐stacks were pre‐processed using ImageJ to normalize the histogram of each slice individually.^[^ ^51^ ^]^ Once imported to Imaris, the “pores” representing the PVA‐T phase of the gels were segmented from the fluorescent GelMA‐RB by using Imaris’ machine‐learning based segmentation (Videos S2 and S3, Supporting Information). To do this, portions of individual Z‐stack slices were manually annotated to denote foreground and background. The machine learning model was trained using manual annotations on sample images representing each preprogrammed delay period. The same model was then used across all Z‐stacks to identify the pores as Surface objects (Figure 3A), as defined within the software. These Surface objects were then used as the basis for a distance transformation algorithm which assigned a value to each pixel within the pores corresponding to how far that pixel was from the Surface border (Figure S4, Supporting Information). In this way, a second channel within the dataset was created, and its maximum value (i.e., the deepest point within a pore for that Z‐stack) was used to numerically compare pores between gels. These maximum values were doubled to serve as a measure of maximum pore diameter and then imported into GraphPad Prism for plotting and descriptive statistics. All analyses were conducted on a Dell Precision T8720 computer with a 16‐core, 3.8 GHz Intel Xeon Gold processor, 256GB DDR4 RAM, and 24GB NVIDIA RTX A5000 GPU running Windows 10.

Additionally, the image channel created by the distance transform algorithm was used to identify Filament objects within the Imaris software. These Filament objects were made up of Segments which correspond to branches of the pore regions (Figure 3B). The software ascribes characteristic properties to each identified segment, such as length and orientation angle. For each Z‐stack, these lengths and orientation angles were exported for analysis using a custom Python script.^[^ ^52^, ^53^, ^54^, ^55^, ^56^ ^]^ The script filtered out all Segments with lengths under 10 µm, as these were deemed to lack real physical meaning in the context of the identified pores. The orientation angles of the remaining Segments were then collected into datasets and analyzed using the Rayleigh test of uniformity.^[^ ^57^ ^]^

### Spatial Tailoring of Pore Size using Dynamic Exposure

In‐order to demonstrate the ability to spatially control pore size across a given exposure area, additional gels were fabricated, here using the low DoF GelMA‐RB combined with the high MW PVA‐T. For these gels, both a 4 mm circle and a 4 mm × 4 mm rectangle were cured. Prior to curing, the prepolymer mixture was acoustically mixed as above. However, instead of one uniform exposure, the gel was broken up into four separate regions. The print head remained stationary, and the exposure in each region was programmed to begin at different times following the acoustic mixing (0, 0.5, 2, or 10 s). Each region was exposed at 140 mW cm^−2^ for 1 s, independently of the other regions. The gels were washed and incubated in DPBS as described previously, then tiled confocal images were acquired to illustrate the change in pore size throughout the XY plane of the gels.

## Conflict of Interest

The authors declare no conflict of interest.

## Supporting information



Supporting Information

Supplemental Video 1

Supplemental Video 2

Supplemental Video 3

## Data Availability

The data that support the findings of this study are available in the supplementary material of this article.
