# Supplementary material for: Tuneable Hydrogel Porosity via Dynamic Tailoring of Spinodal Decomposition
Source: Adv Sci (Weinh). 2025 Jun 26;12(35):e04265. doi: 10.1002/advs.202504265 (PMC12463055; doi:10.1002/advs.202504265)
Supplement: Supplementary file 1 — Supporting Information [file ADVS-12-e04265-s004.docx]

Supplementary Information for Tuneable hydrogel porosity via dynamic tailoring of spinodal decomposition

Michael Halwes^1,2^, Callum Vidler^1,2^, Lilith Caballero Aguilar^1,2,3^, Farzaneh Taromian^1,2,3^, David R Nisbet^1,2,3,4^, Melanie Stamp^1,2^, Khoon S. Lim^2^, Andrea O’Connor^1,2,3^, David J. Collins^1,2^

^1^ *Department of Biomedical Engineering, University of Melbourne, Parkville, VIC 3010, Australia*

^2^*The Graeme Clark Institute, The University of Melbourne, Parkville, Melbourne, VIC 3010, Australia*

^3^*Aikenhead Centre for Medical Discovery, St. Vincent’s Hospital, Melbourne, VIC 3065, Australia*

^4^*Melbourne Medical School, Faculty of Medicine, Dentistry and Health Science, The University of Melbourne, Melbourne, VIC 3010, Australia*

^5^ *Faculty of Medicine and Health, University of Sydney, Sydney, NSW 2006, Australia*

# Results

## Interfacial Tension

A graph of interfacial tensions can be found in **Supp. Fig. 7**. The interfacial tension of the low DoF and high DoF GelMA was 68 and 84 mN/m, respectively, indicating that the higher concentration of methacryloyl groups increased the interfacial tension. Modifying the high MW PVA with tyramine increased the interfacial tension from 57 mN/m to 73 mN/m. Despite differences in molecular weight, the low MW PVA-T showed a yet higher interfacial tension of 84 mN/m. Tyramine has been shown to form hydrogen bonds between the amine and hydroxyl groups of neighboring molecules^32^. These results suggest that increased chain arrangement of the PVA-T molecules is the main contribution to the higher interfacial tension.

## Evaluating Differences in Phase Separation Behavior

When observing the pore morphology of the gels, the mixture of high DoF GelMA with PVA-T began to separate via nucleation at an earlier time point than low DoF GelMA with PVA-T (**Fig 2A**). As our aim was to present a method by which pore architecture could be tailored within hydrogels, a quantitative analysis of the differences in polymer-polymer interactions is outside the scope of this work. However, by considering Flory-Huggins solution theory, a qualitative explanation can be given.

Since the composition and temperature of the two cases are identical, the Flory-Huggins interaction parameter, χ, can be regarded to evaluate the difference in behavior. The Flory-Huggins parameter is a dimensionless quantity that characterizes the interactions of both polymer-solvent and polymer-polymer pairs:

$$\chi=\frac{z}{k_{B}T}\left( \omega_{12}-\frac{\omega_{11}-\omega_{22}}{2} \right),$$

Where $z$is the coordination number of the lattice being considered, $k_{B}$ is Boltzmann’s constant, $T$ is temperature, and $\omega_{12}, \omega_{11}$, and $\omega_{22}$ are the pairwise interaction energies of the polymer species with each other and themselves, respectively^39^. The composition (i.e., the volume fraction) and interaction parameter of a given polymer blend determines whether the system will be stable, unstable, or metastable. These states determine whether the solution will remain homogenously mixed (stable) or separate (i.e. demix) via spinodal decomposition (unstable) or nucleation (metastable).

If demixing is energetically favorable (corresponding to positive values of χ), an initially homogenous mixture will separate until two phases exist whose compositions lie on the binodal curve, defined as the phase boundary between a one-phase and two-phase system^39^. Interestingly, both high and low DoF GelMA mixtures initially undergo spinodal decomposition, meaning the system starts in an unstable state. As the composition of the two phases diverge, the system must first enter the metastable state before reaching equilibrium. The time at which the system transitions from spinodal decomposition to nucleation depends on the magnitude of χ and therefore the relative magnitudes of the $\omega_{12}, \omega_{11}$, and $\omega_{22}$ terms.

The higher concentration of methacryloyl groups in the high DoF GelMA + PVA-T mixtures would have led to more hydrogen bonds between GelMA chains. This is equivalent to saying the high DoF GelMA would have had a higher pairwise interaction energy with itself, reducing the χ value for that system. This in turn led to the high DoF GelMA + PVA-T mixture transitioning to nucleation earlier than the low DoF GelMA + PVA-T. Of course, these speculations would need to be confirmed by more in-depth investigations into the thermodynamic properties of these polymer blends.

# Supplementary Videos

Supplementary Video 1 depicts the fabrication process for the High DoF GelMA + PVA gel, including the acoustic mixing, delay period, and light exposure for curing.

Supplementary Videos 2 and 3 depict rotating views of the 3D reconstructions of High DoF GelMA + PVA-T gels as a representative depiction of the output of the image segmentation process. Video 2 shows the reconstruction for a gel in which the delay period was 0.5 s, and Video 3 shows the same for a gel in which the delay period was 10 s. The main view shows the volume that corresponded to the porogen phase, while the upper right cutout shows the raw Z-stack from confocal imaging (shown in red) combined with the distance transform for the porogen phase (shown in blue). In Video 2, the scale bar (lower left corner) represents 30 μm, and in Video 3, the scale bar represents 50 μm. In both videos, 3D volumes with different colors represent distinct, disconnected objects.

# Supplementary Figures


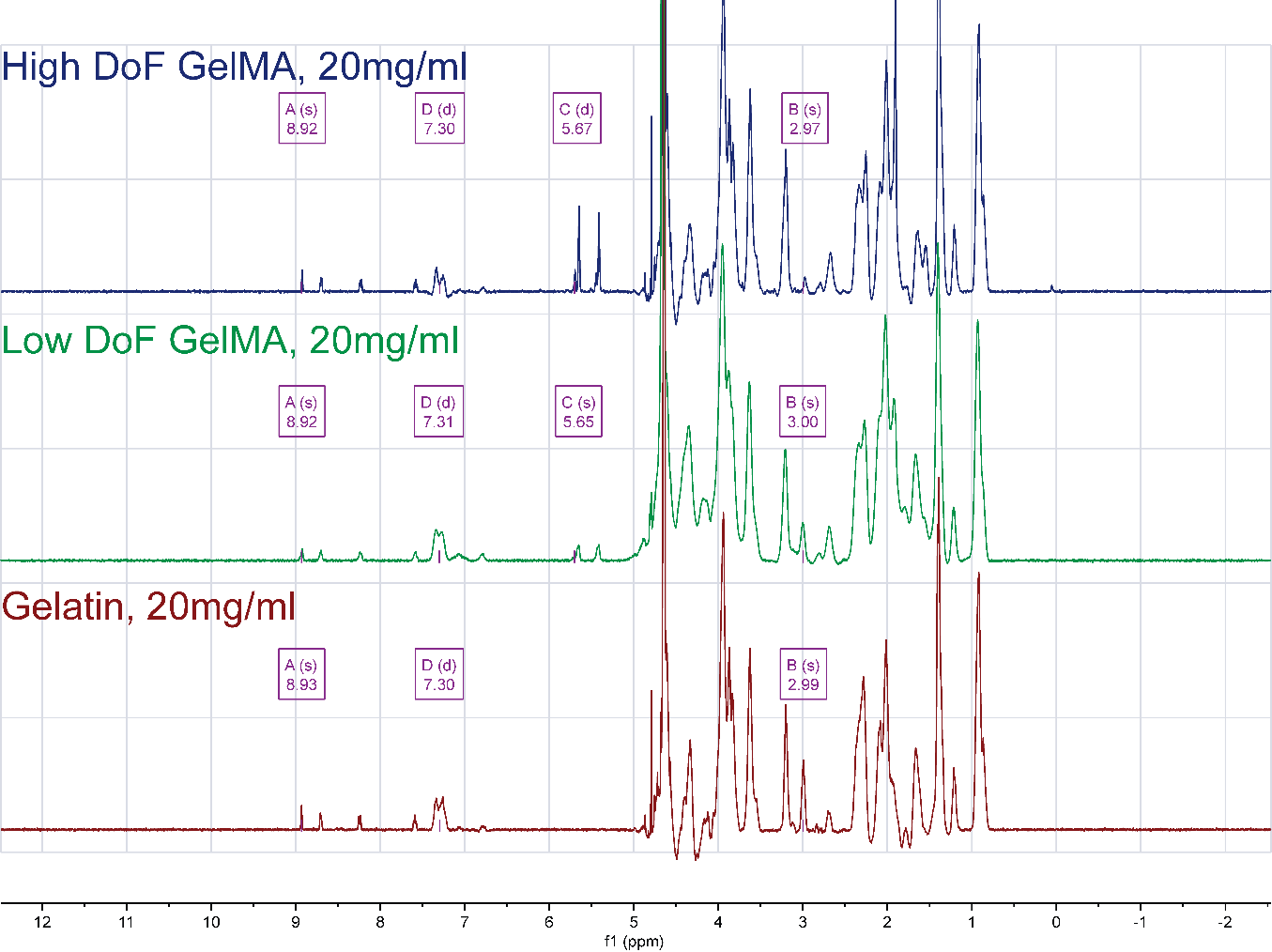


**Supplementary Figure S1:** NMR spectra of unmodified gelatin and the two batches of gelatin methacryloyl synthesized for the present work. Purple labels in the spectra denote the peaks used to determine the degree of functionalization: A – nicotinamide standard, B – lysine, C – methacryloyl groups, D – aromatic groups on gelatin.


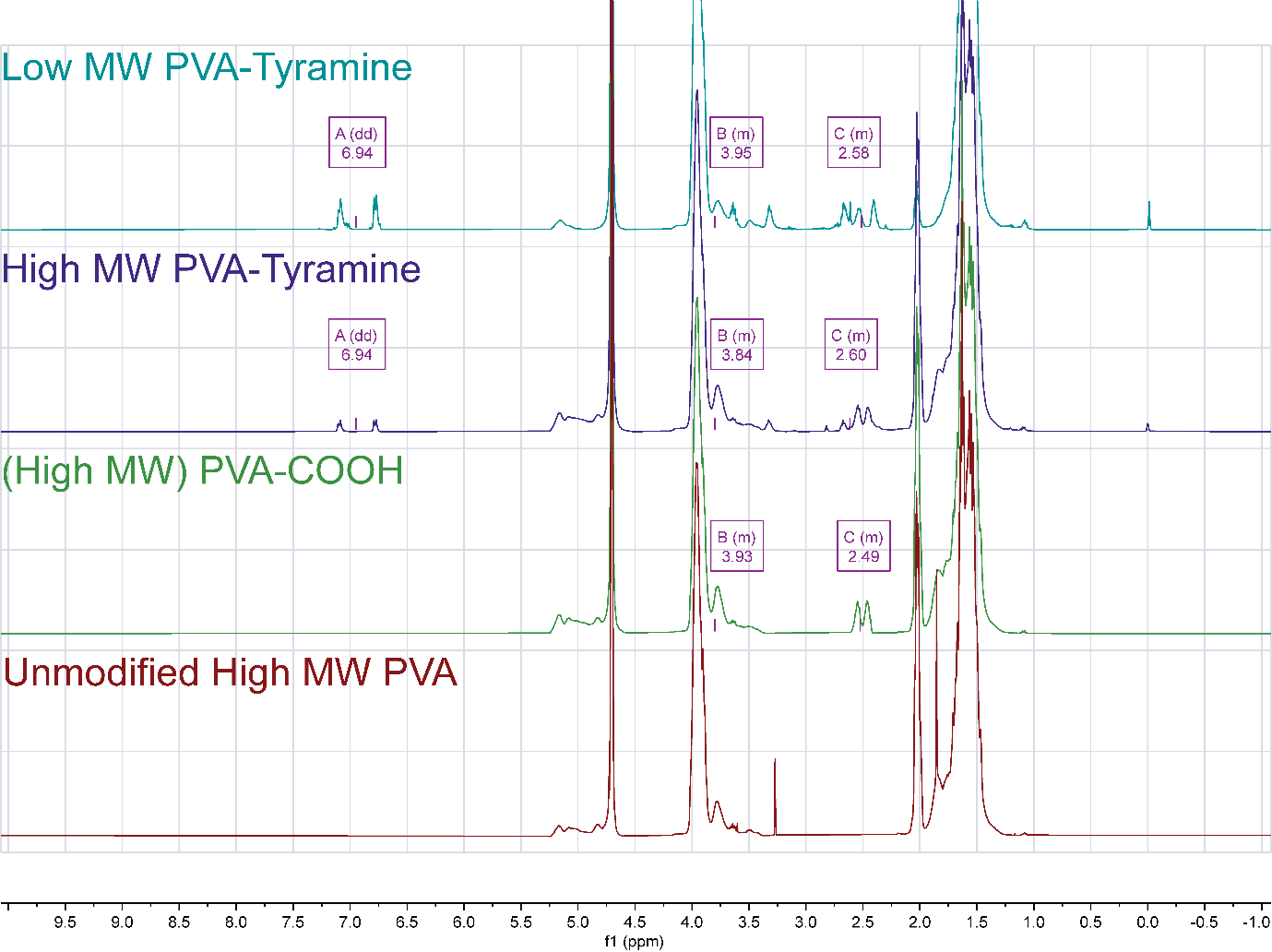


**Supplementary Figure S2:** NMR spectra of Unmodified PVA, carboxylated PVA (PVA-COOH), High MW PVA-Tyramine, and Low MW PVA-Tyramine. The PVA-COOH and High MW PVA-Tyramine were synthesized using the unmodified PVA (87-89% hydrolyzed, 85-124 kDa) shown, whereas the Low MW PVA-Tyramine was synthesized using a 99% hydrolyzed, 18 kDa MW PVA. Purple labels in the spectra denote the peaks used to determine the degree of conjugation: A – aromatic protons on tyramine, B – methylene protons on PVA backbone, C – methylene protons on carboxyl group. The peaks at approx. 3.25 ppm and 1.8 ppm in the unmodified PVA spectra were presumed to be impurities from the manufacturer.


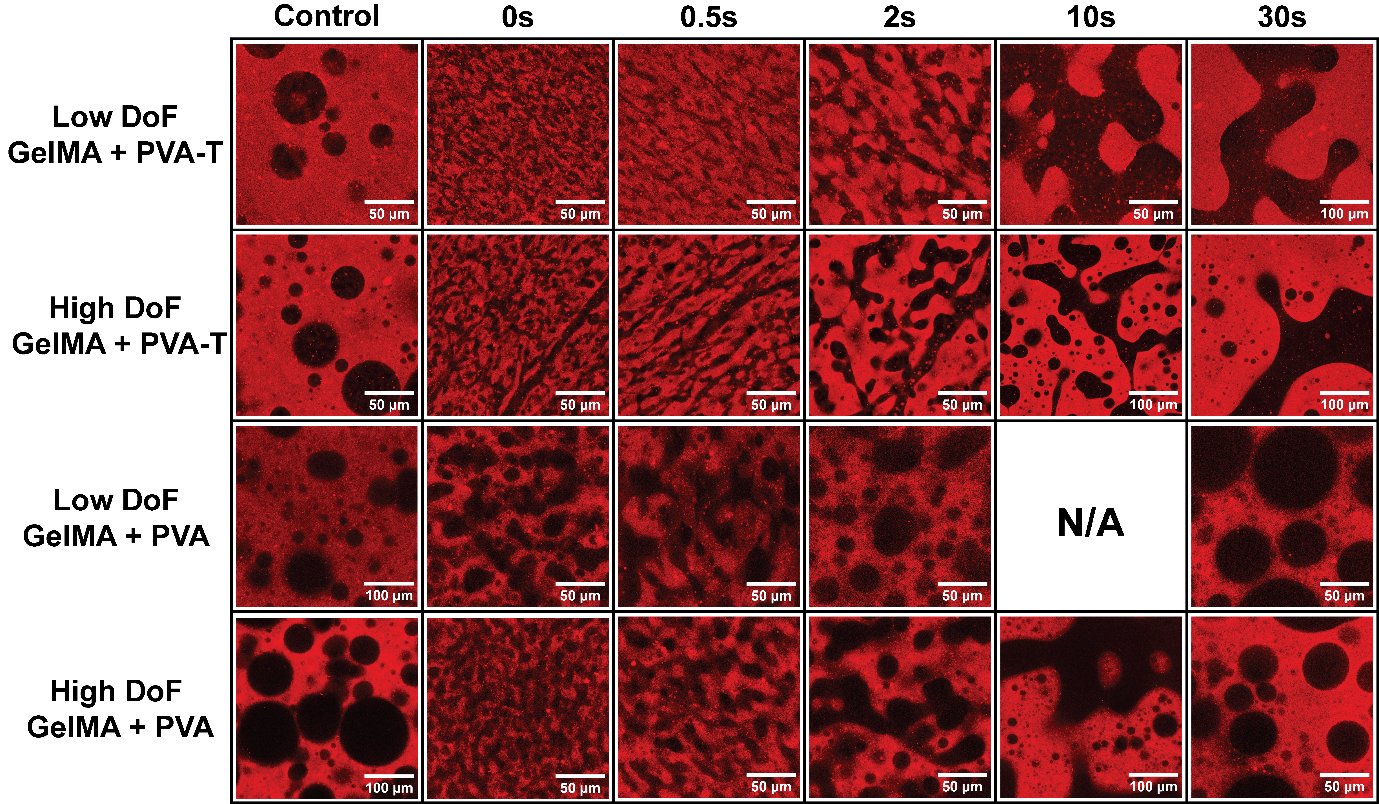


**Supplementary Figure S3:** Sample confocal images from Z-stacks taken from all combinations of gels.


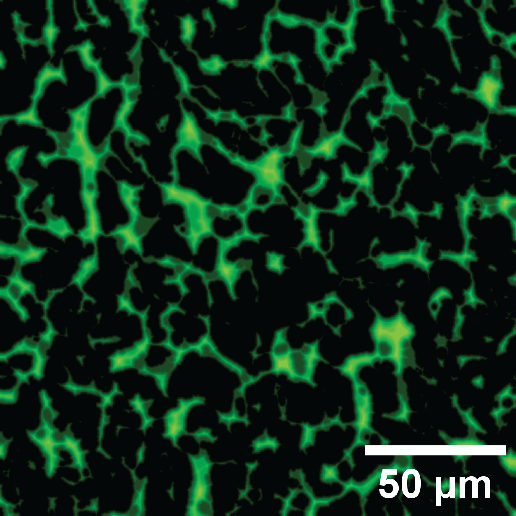


**Supplementary Figure S4:** Sample 2D image taken from the distance transform of the reconstructed pore network. Here, a higher intensity corresponds to higher depth within the pore.


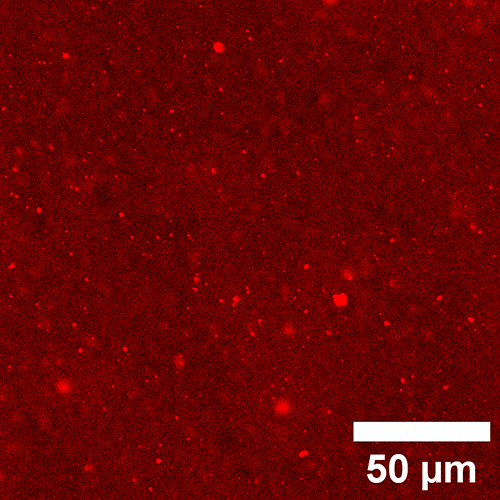


**Supplementary Figure S5:** Confocal image taken from a gel containing the low MW PVA-Tyramine, cured 30 s after acoustic mixing had ceased, indicating a lack of phase separation.


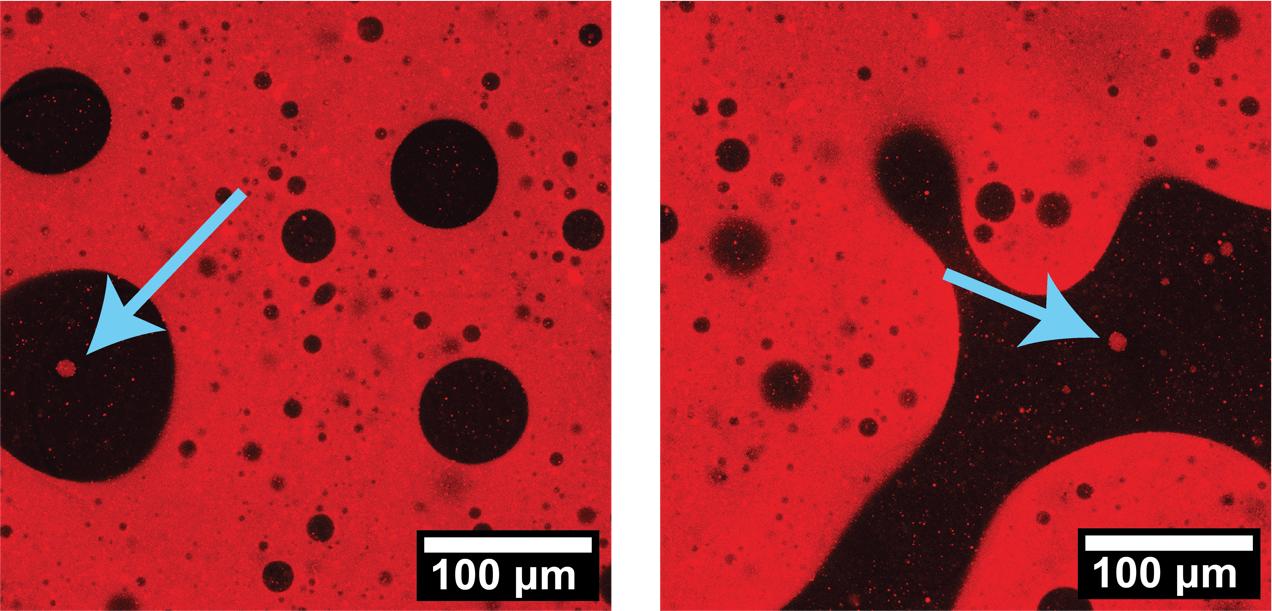


**Supplementary Figure S6:** Confocal images showing droplets of GelMA-RB encapsulated within the PVA-Tyramine phase of the gel mixtures. Both images are taken from Z-stacks acquired in gels which were cured 30 s after the acoustic mixing had ceased.


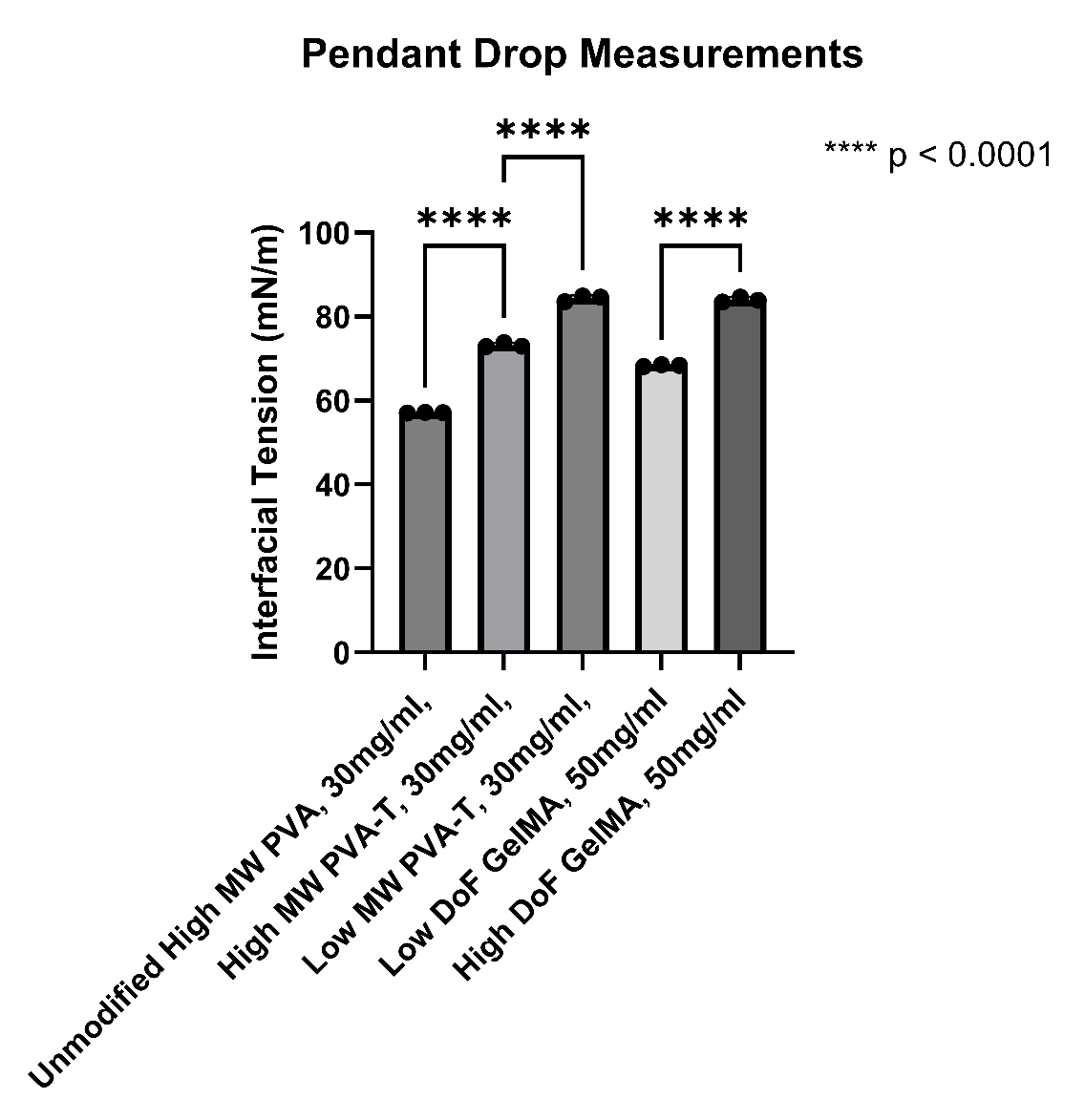


**Supplementary Figure S7:** Tensiometer measurements for the components of the mixtures used to create the aqueous two-phase emulsion hydrogels.


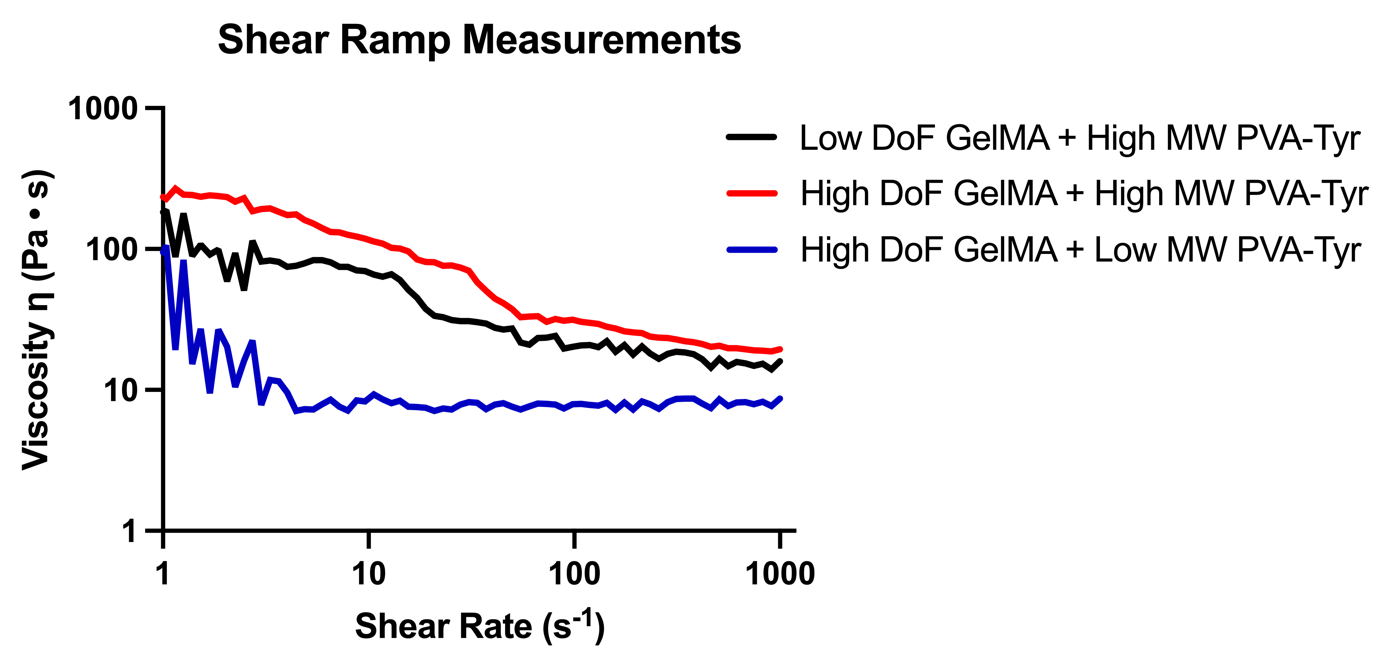


**Supplementary Figure S8:** Rheometer measurements showing the viscosity of the mixtures used to create the aqueous two-phase emulsion hydrogels. The concentrations used for these samples matched those used in the acoustic mixing experiments: 50 mg/ml for GelMA, 30 mg/ml for PVA-T.


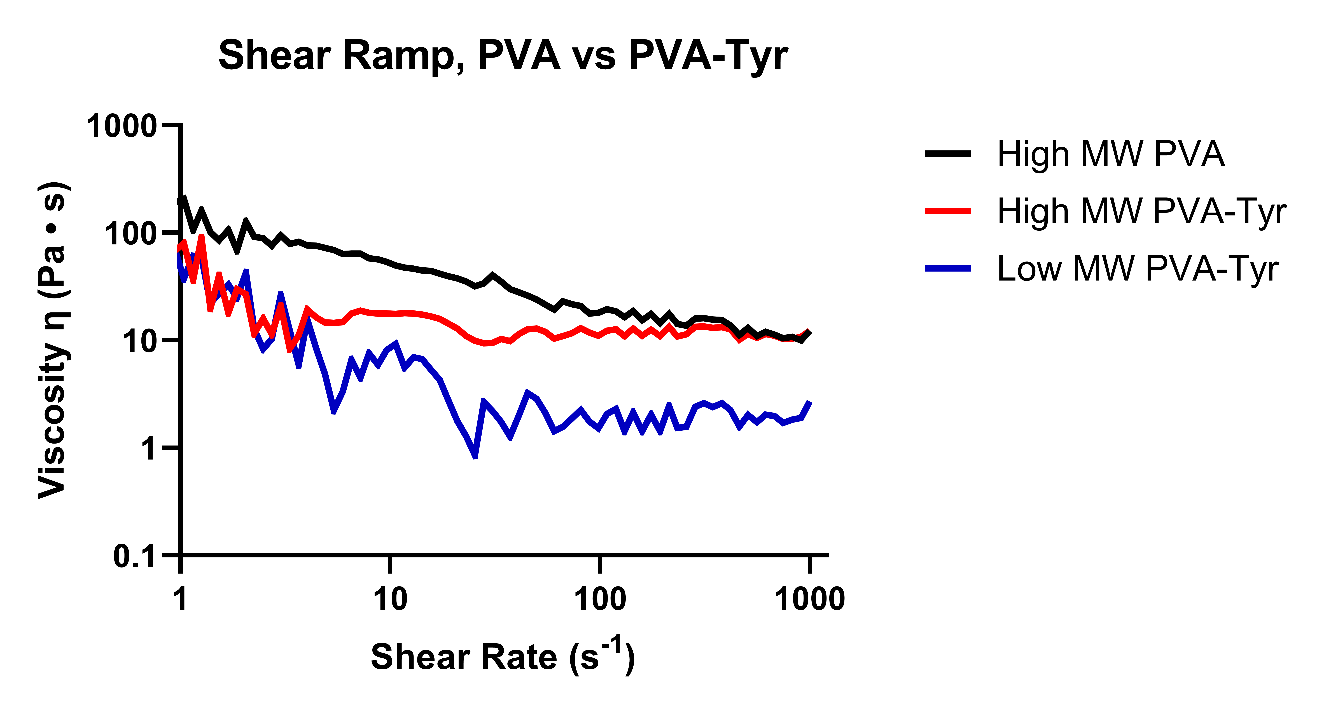


**Supplementary Figure S9:** Rheometer measurements showing the viscosities of the modified and unmodified PVAs used in this study. All samples were tested at 30 mg/ml concentration.
